# Effects of curcumin and ursolic acid in prostate cancer: A systematic review

**DOI:** 10.1177/03915603231202304

**Published:** 2023-09-30

**Authors:** Benjamin D Besasie, Achinto Saha, John DiGiovanni, Michael A Liss

**Affiliations:** 1 Department of Urology, University of Texas Health San Antonio, San Antonio, TX, USA; 2 Division of Pharmacology and Toxicology, College of Pharmacy, The University of Texas at Austin, USA; 3 Department of Urology, South Texas Veterans Healthcare System, USA

**Keywords:** Curcumin, diarylheptanoid, flavonoid, NF-κB, pentacyclic triterpenoid, phytonutrients, prostate cancer, triterpenoid, ursolic acid

## Abstract

The major barriers to phytonutrients in prostate cancer therapy are non-specific mechanisms and bioavailability issues. Studies have pointed to a synergistic combination of curcumin (CURC) and ursolic acid (UA). We investigate this combination using a systematic review process to assess the most likely mechanistic pathway and human testing in prostate cancer. We used the PRISMA statement to screen titles, abstracts, and the full texts of relevant articles and performed a descriptive analysis of the literature reviewed for study inclusion and consensus of the manuscript. The most common molecular and cellular pathway from articles reporting on the pathways and effects of CURC (*n* = 173) in prostate cancer was NF-κB (*n* = 25, 14.5%). The most common molecular and cellular pathway from articles reporting on the pathways and effects of UA (*n* = 24) in prostate cancer was caspase 3/caspase 9 (*n* = 10, 41.6%). The three most common molecular and cellular pathway from articles reporting on the pathways and effects of both CURC and UA (*n* = 193) in prostate cancer was NF-κB (*n* = 28, 14.2%), Akt (*n* = 22, 11.2%), and androgen (*n* = 19, 9.6%). Therefore, we have identified the potential synergistic target pathways of curcumin and ursolic acid to involve NF-κB, Akt, androgen receptors, and apoptosis pathways. Our review highlights the limited human studies and specific effects in prostate cancer.

## Background

Prostate cancer (PCa) is the most common non-cutaneous cancer in American men. It is the second leading cause of cancer-related death in men, with 248,530 estimated new diagnoses in 2021.^1^ The majority of men are diagnosed with low-risk prostate cancer, and guidelines currently emphasize close monitoring. Despite the patient anxiety associated with diagnosis, there are no drugs currently used to slow the prostate cancer progression, specifically for men on active surveillance.^2^

According to the 2022 AUA/ASTRO guidelines, men with clinically localized PCa as defined by clinical stage <T3 without nodal or distant metastasis on conventional imaging can be considered as candidates for active surveillance management in conjunction with individualized risk stratification including clinical T stage, serum prostate-specific antigen (PSA), Grade Group (Gleason score), and tumor volume.^3^ Therefore, due to the wide criteria including multiple clinical stages for men on active surveillance (up to stage T3), the large and clinically diverse patient load provides opportunity for new or additional therapy at varying stages. Active surveillance is a strategy to postpone immediate therapy with the option of delayed intervention in men with low-risk or with low volume favorable intermediate risk. Typically, the follow-up includes serial PSA blood tests, exams, and MRI imaging with repeated biopsy.^4^ The rate of clinical progression and need for treatment for patients on active surveillance is approximately 50% over 5 years, and there are limited strategies to reduce progression.^5^ Moreover, while there is no universally accepted active surveillance protocol, effective therapies, with limited side effect profiles based on scientifically justified rationales, such as phytochemicals, are urgently needed for the majority of men diagnosed with prostate cancer, due to the rate of progression and even more so for those at a higher clinical stage on active surveillance.

Dietary supplements, such as curcumin (CURC) and ursolic acid (UA), are utilized in several anti-inflammatory conditions, largely targeting NF-κB, AKT, and STAT3, common cancer molecular pathways.^6^ Investigators have suggested a variety of dietary and natural products with therapeutic benefits for prostate cancer, yet few have undergone prospective testing in humans. In a landmark study, researchers systematically discovered and proved the synergistic effect of combining the phytochemicals curcumin and ursolic acid in slowing the growth of prostate cancer.^7^ We acknowledge current clinical trials investigating CURC alone in various stages of prostate cancer (NCT03769766, NCT02724618, NCT02064673, NCT03211104); however, a synergistic approach using CURC and UA may provide a more efficacious treatment option than curcumin alone. In addition to their effects, the bioavailability of these supplements raises another issue through poor absorption, rapid metabolism, and rapid systemic elimination.^8,9^ Formulations including liposomes, nanoparticles, phospholipid complexes, structural analogs and derivatives have been investigated to overcome this hurdle.

Therefore, we performed a systematic review of research studies involving prostate cancer and phytochemicals, CURC and UA, by assessing their outcomes on absorption and bioavailability and pathways and effects. Upon search, there have been no systematic reviews involving both CURC and UA in relation to prostate cancer. By investigating these phytochemicals, our hope is that the results of this review will elucidate information for others to perform future clinical trials in patients with prostate cancer, specifically those on an active surveillance regimen and/or monitoring low-grade prostate cancer.

## Methods

### Protocol registration

We used the Preferred Reporting Items for Systematic Reviews and Meta-Analyses (PRISMA) statement, which is an evidence-based minimum set of items reporting in systematic reviews.^10^ We registered the protocol with the International Prospective Register of Systematic Reviews (PROSPERO), registration number: 2020 CRD42020202069.

### Eligibility criteria

We performed a literature review search using PubMed database encompassing all years of publications. We also gathered literature from reference lists from our relevant institutional studies and protocols without performing forward or backward citations. We excluded meta-analyses and reviews. We followed the PICO tool through the Cochrane Handbook for our inclusion criteria. For population, we included all studies with prostate cancer, in vitro and in vivo (both animal and human). For intervention we included phytonutrients curcumin and ursolic acid and any analogs. For comparison, we included placebo, or standard of care medications, or none-reported. For outcomes, we included those reporting on absorption or bioavailability and pathways or effects.

### Information sources

We reviewed only published articles on PubMed database in English and performed no further communication with other study authors. Other than the previously mentioned relevant reference lists, no additional datasets were used or obtained for this review.

### Search

In December 2019, we performed a comprehensive search without a set publication timeframe using the following search terms as our primary variables: “prostate cancer” and “curcumin” or “prostate cancer” and “ursolic acid.” We did not use any secondary variable search terms, such as the phytonutrients’ umbrella chemical compound classes “diarylheptanoid” or “flavonoid” for curcumin or “pentacyclic triterpenoid” or “triterpenoid.”

### Study selection

We excluded all meta-analyses and reviews from the final list. We divided the literature into two broad categories: absorption or bioavailability and pathways or effects.

### Data collection process

**Figure 1.**
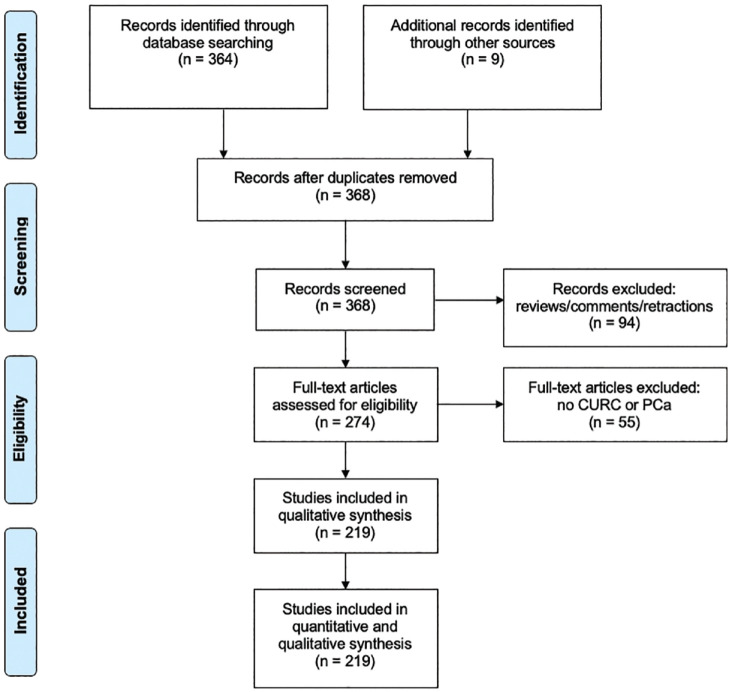
PRISMA flow diagram of literature search involving **curcumin** and prostate cancer.

**Figure 2.**
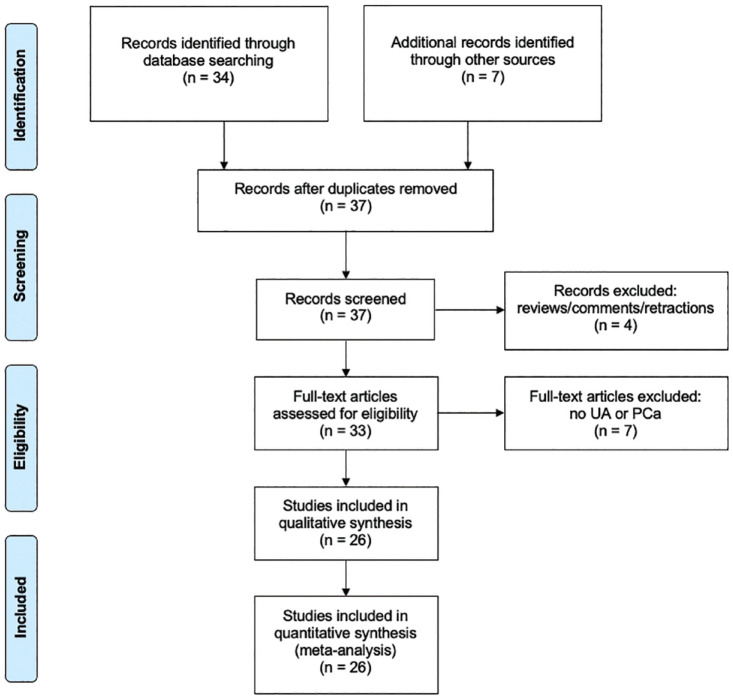
PRISMA flow diagram of literature search involving **ursolic acid** and prostate cancer.

We display our data collection processes in Figures 1 and 2. One person (BDB) conducted data extraction for consistency.

### Data items

Search terms were used in PubMed only. We did exclude studies not directly related to prostate cancer.

### Risk of bias in individual studies

For human studies, we noted prospective, retrospective, or observational studies to be transparent about the strength of associations inferred from the inherent bias of study design. Additionally, for any human studies, we added the sample size of each study to provide the reader with information regarding enrollment. We did not report on publication bias.

### Summary measures

We divided tables into a study outcomes group, in vitro and in vivo studies, while further dividing into animal and human studies. For both curcumin and ursolic acid separately, we also reported data on year of publication and country of origin. Furthermore, we provided summary measures for human studies involving curcumin only. Finally, we reported most common molecular and cellular pathways and bioavailability delivery methods for both CURC and UA in prostate cancer.

### Synthesis of results

We performed a descriptive analysis of the literature reviewed for study inclusion and consensus of the manuscript. After initial review and selection of articles, we performed a qualitative data analysis, using the computer program NVivo (QSR International, LLC, Massachusetts, US), to find common conclusions and relationships. We did not use further statistical analysis.

### Risk of bias across studies

A qualitative assessment was performed using the guidelines for assessing quality in the research based on potential biases.^11^ We included some aspects of the confounders and outcome measurements within the summary tables.

## Results

### Overall search results

We located a total of 373 CURC-related studies and 41 UA-related studies in the initial search spanning from publication years 2000–2020. We present the PRISMA flow diagram for our search strategy in Figure 1 for CURC and Figure 2 for UA.

**Figure 3.**
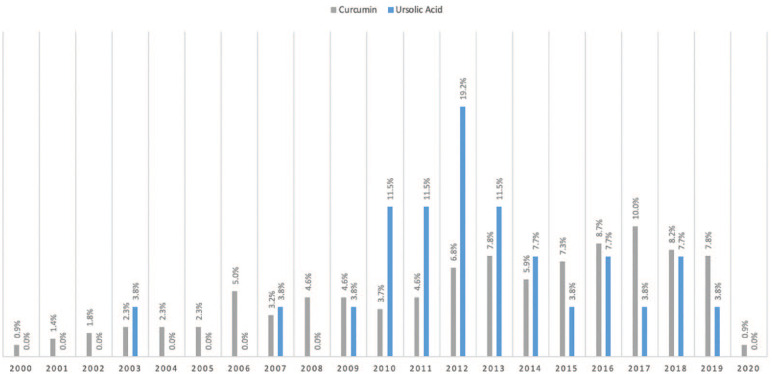
Year of study published involving **curcumin** (*n* = 219) and **ursolic acid** (*n* = 26) and prostate cancer. Counts and percentage of publication years can be found in Supplemental Table 1.

**Figure 4.**
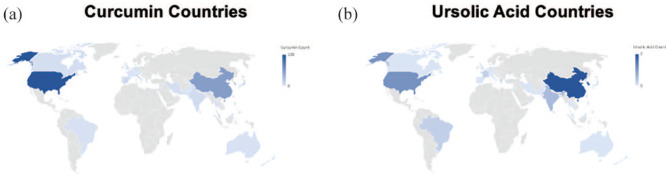
Countries of origin world map distribution of published studies involving: (a) **curcumin** and (b) **ursolic acid** and prostate cancer. *Seven articles on curcumin reported more than one country of origin. Counts and percentage of publication countries of origin can be found in Supplemental Table 2.

Overall, 219 studies involved CURC and PCa, while 26 studies investigated UA and PCa, with the majority focusing on molecular pathways and effects rather than absorption and bioavailability. No reviewed studies involved both CURC and UA combined. We have outlined particular groups of articles based on absorption/bioavailability, pathways/effects, in vitro, in vivo, or human (Table 1). For both phytonutrients in prostate cancer, the majority of studies were focused in vitro, 198 with CURC and 24 with UA. Alternatively, limited studies were focused in vivo, 61 with CURC and 6 with UA. Notably, there were eight (8/219, 3.7%) human studies investigating CURC effect in prostate cancer in contrast to no human studies investigating UA effect in prostate cancer. More specifically, a qualitative summary of the eight CURC-related human studies is presented in Table 2, with results focusing mainly on PSA effects.^12–19^ We display the number of published articles by year in Figure 3 demonstrating a steady and increasing publication record since 2000 more so for CURC with the largest number of publications in 2017 for CURC (*n* = 22) and 2012 for UA (*n* = 5). Only two studies reported publication in 2000 since our search was initiated in late 2019. We also display the number of published articles by country of origin in Figure 4 demonstrating a majority publication record by the United States (*n* = 100) for CURC and China (*n* = 7) for UA.

**Table 1.** Categorization of reviewed articles relating to **curcumin** or **ursolic acid** and prostate cancer.

Phytonutrient	Total	Absorption & bioavailability	Pathways & effects	In vitro	In vivo	In vivo (Human)
Curcumin	219	58^a^	173^a^	198^b^	61^b^	8
Ursolic Acid	26	4^a^	24^a^	24^b^	6^b^	0
Total	245	62	197	222	67	8

a 12 articles on curcumin and 2 articles on ursolic acid reported data on both Absorption & Bioavailability and Pathways & Effects outcomes categories.

b 40 articles on curcumin and 5 articles on ursolic acid conducted studies both in vitro or in vivo. One article on ursolic acid did not report whether study was conducted in vitro or in vivo.

**Table 2.** Human studies evaluating the effects of **curcumin** (*n* = 8) in prostate cancer.^a^

	Study ID	Year	Study type	Randomized	Inclusion criteria	Study size	Intervention	Curcumin dose	Time treated	Comparisons	Outcomes category	Outcomes	Safety
12	Choi YH, PMID: 30671976	2019	Prospective	Yes	PCa with intermittent androgen deprivation	91 (1:1)	Curcumin	1440 mg/day	6 months	Placebo	Pathways and effects	PSA elevation suppressed	Safe and well tolerated
13	Saadipoor A, PMID: 30427093	2019	Prospective	Yes	PCa and candidate for IMRT	64 (1:1)	Nanocurcumin	120 mg/day	3 days before and during RT	Placebo	Pathways and effects	No outcome difference detected	Well tolerated
14	Greil R, PMID: 30074076	2018	Prospective	No	Metastatic cancer	32 (2 PCa)	Lipocurc™ (liposomal curcumin)	100–300 mg/m^2^	8 weeks	None	Absorption and bioavailability	Maximum tolerated dose	Well tolerated
15	Ledda A, PMID: 29028078	2017	Prospective	No	PCa or bladder cancer	61 (26:35)	Oncotris (curcumin, cordyceps, and astaxanthin)	Unknown	6 weeks	Placebo	Pathways and effects	PSA reduced	No adverse effects
16	Ried K, PMID: 28843267	2017	Observational	N/A	PCa (or other cancer)	542 (5 PCa)	Curcumin	Unknown	Unknown	None	Pathways and effects	CTC count reduction	No adverse effects
17	Mahammedi H, PMID: 26771576	2016	Prospective	No	Castration-resistant PCa with rising PSA	26	Curcumin/docetaxel/prednisone	6000 mg/day	6 days	None	Pathways and effects	PSA reduction	No adverse effects and well tolerated
18	Hejazi J, PMID: 26771294	2016	Prospective	Yes	PCa with EBRT	40 (1:1)	Curcumin	3000 mg/day	During RT	Placebo	Pathways and effects	PSA elevation suppressed	No adverse effects
19	Ide H, PMID: 20503397	2010	Prospective	Yes	Negative prostate biopsy with no PIN	85 (1:1)	Curcumin/isoflavones	100 mg	6 months	Placebo	Pathways and effects	PSA reduced	No adverse effects

a There are no studies investigating the impact of ursolic acid on prostate cancer.

#### Curcumin mechanism in prostate cancer

**Figure 5.**
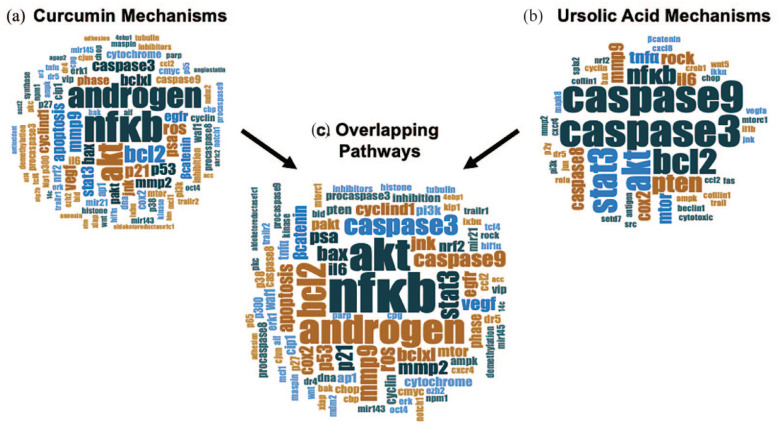
Word cloud of curcumin and ursolic acid mechanistic pathways in prostate cancer individually and combined. (a) Word cloud of most common molecular and cellular pathways from articles reporting on the pathways and effects of **curcumin** (*n* = 173) in prostate cancer. Larger words signify greater overlap and common pathways across all articles. Counts and percentage of most common pathways can be found in Supplemental Table 4. Two articles (1.2%) did not report any specific effects. (b) Word cloud of most common molecular and cellular pathways from articles reporting on the pathways and effects of **ursolic acid** (*n* = 24) in prostate cancer. Larger words signify greater overlap and common pathways across all articles. Counts and percentage of most common pathways can be found in Supplemental Table 6. (c) Word cloud of most common molecular and cellular pathways from articles reporting on the pathways and effects (*n* = 197) of both **curcumin** (*n* = 173) and **ursolic acid** (*n* = 24) in prostate cancer. Larger words signify greater overlap and common pathways across all articles. Counts and percentage of most common pathways can be found in Supplemental Table 7.

We identified 373 CURC-related studies with 5 duplicate articles and 94 reviews/comments/retractions were removed. Of the 274 articles remaining, 55 studies were excluded upon eligibility assessment due to no reference to CURC or PCa. Finally, 173 of the 219 articles relating to CURC and PCa reported data outcomes on the mechanistic pathways (Supplemental Table 3), which we used to perform descriptive and qualitative analysis.^7,12,13,15–184^ The word cloud of the most common pathways affected in prostate cancer is displayed in Figure 5(a) and Supplemental Table 4, with the pathway involving NF-κB as the most common (*n* = 25/173, 14.5%).

#### Ursolic acid mechanism in prostate cancer

We identified 41 UA-related studies with four duplicate articles and four reviews were removed. Of the 33 articles remaining, 7 studies were excluded upon eligibility assessment due to no reference to UA or PCa. Finally, 24 of the 26 articles relating to UA and PCa reported data outcomes on the mechanistic pathways (Supplemental Table 5), which we used to perform descriptive and qualitative analysis.^7,185–207^ The word cloud of the most common pathways affected in prostate cancer is displayed Figure 5(b) and Supplemental Table 6, with the pathway involving caspase 3/caspase 9 as the most common (*n* = 10/24, 41.7%). Specific to humans, five prospective studies reported curcumin effects on prostate-specific antigen (PSA) reduction.

#### Combination of curcumin and ursolic acid

In order to identify the best pathway described in the literature using both compounds we performed a third word cloud analysis (Figure 5(c)) with the combination of all articles within the review that reported data outcomes on mechanistic pathways (*n* = 197; CURC = 173, UA = 24). The most common combined mechanistic pathways affecting prostate cancer for both CURC and UA involved NF-κB (*n* = 28/197, 14.2%), Akt (*n* = 22/197, 11.2%), and androgen (*n* = 19/197, 9.6%) (Supplemental Table 7).

#### Curcumin bioavailability and prostate cancer

Upon review of study outcome categories for CURC-related studies, a total of 58 (58/219, 26.5%) articles evaluated the absorption or bioavailability^14,27,29,32,38,57,73,76,81,84,87,90,135,208–252^ (Supplemental Table 8). Many studies investigated the delivery of curcumin to increase absorption by using nanoparticles or nanocapsules (*n* = 27/58, 46.6%) or liposomes (*n* = 6/58, 10.3%). While one human study assessed the pharmacokinetics of curcumin, specifically liposomal curcumin, in the blood (Table 2),^10^ none of the eight human studies measured or reported curcumin uptake into normal or cancerous prostate cells. However, all eight human studies investigated varying curcumin dosing regimens from 100 to 6000 mg/day spanning timeframes from 6 days to 6 months in men with prostate cancer (Table 2).

#### Ursolic acid bioavailability and prostate cancer

Upon review of study outcome categories for UA-related studies, a total of 4 (4/26, 15.4%) articles evaluated the absorption or bioavailability^197,201,253,254^ (Supplemental Table 9). Only one study investigated the delivery of ursolic acid to increase absorption by using liposomes (*n* = 1/4, 25%).^254^ In contrast to curcumin, there were no articles identified involving human ursolic acid bioavailability in men with prostate cancer.

## Discussion

Our systematic review and qualitative analysis noted NF-κB as the primary target to investigate the synergistic mechanism of action of curcumin and ursolic acid in prostate cancer. Nuclear factor-kappa B (NF-κB) is a transcription factor essential for inflammatory responses by activating multiple downstream targets^255^ and is now a known cancer therapeutic target.^256^ NF-κB has an established communication pathway with androgen receptors, which agrees with our qualitative analysis assessment.^257^ In addition to androgens as a driver of prostate cancer, NF-κB participates in inflammatory cross-talk, which also is a driver of prostate cancer progression.^258,259^ Additionally, NF-κB is implicated in prostate cancer progression to androgen independence in advanced cancer.^260,261^ From our review, we understand that curcumin and ursolic acid are known to reduce the inflammatory pathway in prostate cancer, likely through NF-κB. Unfortunately, various barriers continue to exist regarding the translation of these compounds as a therapeutic option in prostate cancer.

Despite the growing interest in using dietary supplements for cancer, drug bioavailability remains a major barrier of using natural products for adjunctive cancer therapy. A report in 2009 noted 63% of prostate cancer patients surveyed (*n* = 827) reported using dietary supplements.^262^ In 1994, the Dietary Supplement Health and Education Act gave the FDA the authority to regulate dietary supplements, which did not require pre-market approval, yet does allow the FDA to investigate a supplement for health safety or false claims.^263^ Therefore, dietary supplements can be approved by only showing safety, while efficacy is de-emphasized. Patients commonly request dietary supplements as potential adjuvant therapy, but the research severely lacks test efficacy, particularly in prostate cancer. For example, we investigated human studies of curcumin (*n* = 8) and ursolic acid (*n* = 0) compared to over 200 such studies using animals or cell lines. While some groups are investigating the delivery of curcumin in PCa cell lines using nanoparticles and liposomes, we did not identify any studies investigating the uptake of these phytonutrients specifically in prostate cancer cells in humans. Shown through our review with the majority of studies investigating the molecular mechanisms of these phytonutrients in prostate cancer and becoming increasingly uncovered, more data on bioavailability is now important. Based on the promising data from Lodi et al.,^7^ a translational science approach using new drug development techniques is needed to enhance curcumin and ursolic acid absorption, show targeted mechanism effects, and prove efficacy in human prostate cancer patients.

Other highly ranked molecular pathways from our analysis included Akt (protein kinase B), androgen receptor, and apoptosis pathways (Figure 5). While the androgen receptor is highly studied and known in prostate cancer progression and current treatment, Akt plays a role in the PI3K/AKT pathway of tumorigenesis including apoptosis and proliferation of PCa cells as a part of the NF-κB axis.^264^ Therefore, with the Akt pathway shown to be studied in CURC and UA through our review in combination with NF-κB, these pathways should be further investigated to understand the exact axis points of the effects from CURC and UA. More importantly, these pathways should also be investigated within the tissues of human prostate cancers after exposure to curcumin and ursolic acid combined for their possible synergism.

We acknowledge the limitations of this study in that not all the studies are performed in a similar manner. We used a novel technique for analysis of our systematic review because the nature of non-human basic science does not allow for standard tools for effect size. Using the primary findings from the abstracts or results section, we utilized the NVivo software to identify the most common terms. Also, the pathway studies were not random in many manuscripts. Investigators usually choose a particular pathway to investigate and because NF-κB is a key pathway for many cancers, this pathway may have been selected more so than other pathways.

## Conclusions

We identified ample in vitro studies involving the effects of CURC with fewer in vitro studies involving the effects of UA in prostate cancer. We use this review to identify a target pathway of the synergistic effects of curcumin and ursolic acid and show that NF-κB and Akt to be the most investigated pathway effect in prostate cancer. Our review highlights the limited human studies and data of these phytonutrients’ bioavailability and effects in prostate cancer. With the lack of possible treatment for men with low-grade prostate cancer, this review identifies areas of investigation on CURC and UA treatment outcomes in prostate cancer human clinical trials.

## Supplemental Material

sj-docx-1-urj-10.1177_03915603231202304 – Supplemental material for Effects of curcumin and ursolic acid in prostate cancer: A systematic review Supplemental material, sj-docx-1-urj-10.1177_03915603231202304 for Effects of curcumin and ursolic acid in prostate cancer: A systematic review by Benjamin D Besasie, Achinto Saha, John DiGiovanni and Michael A Liss in Urologia Journal

sj-docx-2-urj-10.1177_03915603231202304 – Supplemental material for Effects of curcumin and ursolic acid in prostate cancer: A systematic review Supplemental material, sj-docx-2-urj-10.1177_03915603231202304 for Effects of curcumin and ursolic acid in prostate cancer: A systematic review by Benjamin D Besasie, Achinto Saha, John DiGiovanni and Michael A Liss in Urologia Journal

sj-docx-3-urj-10.1177_03915603231202304 – Supplemental material for Effects of curcumin and ursolic acid in prostate cancer: A systematic review Supplemental material, sj-docx-3-urj-10.1177_03915603231202304 for Effects of curcumin and ursolic acid in prostate cancer: A systematic review by Benjamin D Besasie, Achinto Saha, John DiGiovanni and Michael A Liss in Urologia Journal

sj-docx-4-urj-10.1177_03915603231202304 – Supplemental material for Effects of curcumin and ursolic acid in prostate cancer: A systematic review Supplemental material, sj-docx-4-urj-10.1177_03915603231202304 for Effects of curcumin and ursolic acid in prostate cancer: A systematic review by Benjamin D Besasie, Achinto Saha, John DiGiovanni and Michael A Liss in Urologia Journal

sj-docx-5-urj-10.1177_03915603231202304 – Supplemental material for Effects of curcumin and ursolic acid in prostate cancer: A systematic review Supplemental material, sj-docx-5-urj-10.1177_03915603231202304 for Effects of curcumin and ursolic acid in prostate cancer: A systematic review by Benjamin D Besasie, Achinto Saha, John DiGiovanni and Michael A Liss in Urologia Journal

sj-docx-6-urj-10.1177_03915603231202304 – Supplemental material for Effects of curcumin and ursolic acid in prostate cancer: A systematic review Supplemental material, sj-docx-6-urj-10.1177_03915603231202304 for Effects of curcumin and ursolic acid in prostate cancer: A systematic review by Benjamin D Besasie, Achinto Saha, John DiGiovanni and Michael A Liss in Urologia Journal

sj-docx-7-urj-10.1177_03915603231202304 – Supplemental material for Effects of curcumin and ursolic acid in prostate cancer: A systematic review Supplemental material, sj-docx-7-urj-10.1177_03915603231202304 for Effects of curcumin and ursolic acid in prostate cancer: A systematic review by Benjamin D Besasie, Achinto Saha, John DiGiovanni and Michael A Liss in Urologia Journal

sj-docx-8-urj-10.1177_03915603231202304 – Supplemental material for Effects of curcumin and ursolic acid in prostate cancer: A systematic review Supplemental material, sj-docx-8-urj-10.1177_03915603231202304 for Effects of curcumin and ursolic acid in prostate cancer: A systematic review by Benjamin D Besasie, Achinto Saha, John DiGiovanni and Michael A Liss in Urologia Journal

sj-docx-9-urj-10.1177_03915603231202304 – Supplemental material for Effects of curcumin and ursolic acid in prostate cancer: A systematic review Supplemental material, sj-docx-9-urj-10.1177_03915603231202304 for Effects of curcumin and ursolic acid in prostate cancer: A systematic review by Benjamin D Besasie, Achinto Saha, John DiGiovanni and Michael A Liss in Urologia Journal
