# Supplementary material for: Effects of curcumin and ursolic acid in prostate cancer: A systematic review
Source: Urologia. 2023 Sep 30;91(1):90–106. doi: 10.1177/03915603231202304 (PMC10976464; doi:10.1177/03915603231202304)
Supplement: sj-docx-1-urj-10.1177_03915603231202304 – Supplemental material for Effects of curcumin and ursolic acid in prostate cancer: A systematic review [file sj-docx-1-urj-10.1177_03915603231202304.docx]

**Supplementary Table 1.** Number of studies published per year involving **curcumin** (n=219) or **ursolic acid** (n=26) and prostate cancer.

| Year | Curcumin | Ursolic Acid |
| --- | --- | --- |
| 2000 | 2 (0.9%) | 0 (0.0%) |
| 2001 | 3 (1.4%) | 0 (0.0%) |
| 2002 | 4 (1.8%) | 0 (0.0%) |
| 2003 | 5 (2.3%) | 1 (3.8%) |
| 2004 | 5 (2.3%) | 0 (0.0%) |
| 2005 | 5 (2.3%) | 0 (0.0%) |
| 2006 | 11 (5.0%) | 0 (0.0%) |
| 2007 | 7 (3.2%) | 1 (3.8%) |
| 2008 | 10 (4.6%) | 0 (0.0%) |
| 2009 | 10 (4.6%) | 1 (3.8%) |
| 2010 | 8 (3.7%) | 3 (11.5%) |
| 2011 | 10 (4.6%) | 3 (11.5%) |
| 2012 | 15 (6.8%) | 5 (19.2%) |
| 2013 | 17 (7.8%) | 3 (11.5%) |
| 2014 | 13 (5.9%) | 2 (7.7%) |
| 2015 | 16 (7.3%) | 1 (3.8%) |
| 2016 | 19 (8.7%) | 2 (7.7%) |
| 2017 | 22 (10.0%) | 1 (3.8%) |
| 2018 | 18 (8.2%) | 2 (7.7%) |
| 2019 | 17 (7.8%) | 1 (3.8%) |
| 2020 | 2 (0.9%) | 0 (0.0%) |
