# Supplementary material for: Effects of curcumin and ursolic acid in prostate cancer: A systematic review
Source: Urologia. 2023 Sep 30;91(1):90–106. doi: 10.1177/03915603231202304 (PMC10976464; doi:10.1177/03915603231202304)
Supplement: sj-docx-2-urj-10.1177_03915603231202304 – Supplemental material for Effects of curcumin and ursolic acid in prostate cancer: A systematic review [file sj-docx-2-urj-10.1177_03915603231202304.docx]

**Supplementary Table 2.** Number of studies published by country involving **curcumin** (n=219) or **ursolic acid** (n=26) and prostate cancer.

| Country | Curcumin | Ursolic Acid |
| --- | --- | --- |
| Australia | 2 (0.9%) | 0 (0.0%) |
| Austria | 1 (0.4%) | 0 (0.0%) |
| Bahrain | 2 (0.9%) | 0 (0.0%) |
| Brazil | 2 (0.9%) | 1 (3.8%) |
| Canada | 4 (1.7%) | 0 (0.0%) |
| China | 49 (21.4%) | 7 (29.9%) |
| France | 1 (0.4%) | 1 (3.8%) |
| Germany | 2 (0.9%) | 0 (0.0%) |
| Greece | 1 (0.4%) | 1 (3.8%) |
| India | 6 (2.6%) | 2 (7.7%) |
| Iran | 4 (1.7%) | 0 (0.0%) |
| Israel | 1 (0.4%) | 0 (0.0%) |
| Italy | 6 (2.6%) | 0 (0.0%) |
| Japan | 6 (2.6%) | 0 (0 .0%) |
| Jordan | 2 (0.9%) | 0 (0.0%) |
| Korea | 9 (3.9%) | 6 (23.1%) |
| Luxembourg | 2 (0.9%) | 0 (0.0%) |
| Malaysia | 4 (1.7%) | 0 (0.0%) |
| New Zealand | 1 (0.4%) | 0 (0.0%) |
| Pakistan | 1 (0.4%) | 0 (0.0%) |
| Puerto Rico | 1 (0.4%) | 0 (0.0%) |
| Singapore | 0 (0.4%) | 3 (11.5%) |
| Spain | 7 (3.1%) | 0 (0.0%) |
| Switzerland | 1 (0.4%) | 0 (0.0%) |
| Taiwan | 8 (3.5%) | 1 (3.8%) |
| Thailand | 2 (0.9%) | 0 (0.0%) |
| United Kingdom | 3 (1.3%) | 0 (0.0%) |
| United States | 100 (43.7%) | 4 (15.5%) |
| Uruguay | 1 (0.4%) | 0 (0.0%) |
