# Supplementary material for: Effects of curcumin and ursolic acid in prostate cancer: A systematic review
Source: Urologia. 2023 Sep 30;91(1):90–106. doi: 10.1177/03915603231202304 (PMC10976464; doi:10.1177/03915603231202304)
Supplement: sj-docx-3-urj-10.1177_03915603231202304 – Supplemental material for Effects of curcumin and ursolic acid in prostate cancer: A systematic review [file sj-docx-3-urj-10.1177_03915603231202304.docx]

**Supplementary Table 3.** Reviewed articles reporting on the pathways and effects of **curcumin** (n=173) in prostate cancer**.**

|  | Study ID | Participants | Mechanistic Pathway |
| --- | --- | --- | --- |
| 7 | Lodi A,  PMID: 29202102 | In Vitro,  In Vivo (mouse) | ASCT2, STAT3, mTORC1, AMPK |
| 12 | Choi YH,  PMID: 30671976 | In Vivo (human) | PSA |
| 13 | Saadipoor A,  PMID: 30427093 | In Vivo (human) | None reported |
| 15 | Ledda A,  PMID: 29028078 | In Vivo (human) | PSA, blood cell count |
| 16 | Ried K,  PMID: 28843267 | In Vivo (human) | CTC |
| 17 | Mahammedi H,  PMID: 26771576 | In Vivo (human) | PSA |
| 18 | Hejazi J,  PMID: 26771294 | In Vivo (human) | Plasma total antioxidant capacity, superoxide dismutase |
| 19 | Ide H,  PMID: 20503397 | In Vitro,  In Vivo (human) | PSA |
| 20 | Ocasio-Malave C,  PMID: 31767266 | In Vitro | NFκB |
| 21 | Katta S,  PMID: 31581661 | In Vitro | TGFB |
| 22 | Rodriguez Castano P,  PMID: 31533365 | In Vitro | CYP17A1, CYP19A1 |
| 23 | Yang Y,  PMID: 31154107 | In Vitro | Elovl3 |
| 24 | Zhu M,  PMID: 31042325 | In Vitro | miR34a, βcatenin, cmyc |
| 25 | Guo W,  PMID: 30943812 | In Vivo (rat) | Lipid peroxidation, 14C glucose uptakes, 3H-thymidine uptake |
| 26 | Doush Y,  PMID: 30674964 | In Vitro | AGAP2 |
| 27 | Belluti S,  PMID: 30577600 | In Vitro | PI3K, AKT, ERK, p38 |
| 28 | Mapoung S,  PMID: 30499149 | In Vitro,  In Vivo (mouse) | MMP2, MMP9 |
| 29 | Vellampatti S,  PMID: 30297802 | In Vitro | Growth inhibition |
| 30 | Luo J,  PMID: 30248372 | In Vitro | p21 |
| 31 | Fort RS,  PMID: 30110911 | In Vitro | Anti-neoplastic activity |
| 32 | Plyduang T,  PMID: 30062740 | In Vitro | S-phase, p53, p21Waf/CIP1, cyclin D1 |
| 33 | Zhao W,  PMID: 29485738 | In Vivo (mouse) | JNK |
| 34 | Lin W,  PMID: 29425687 | In Vitro,  In Vivo (mouse) | STAT3 |
| 35 | Cheng WL,  PMID: 29374713 | In Vitro | Maspin |
| 36 | Ide H,  PMID: 29369461 | In Vitro,  In Vivo (mouse) | CYP11A1, HSD3B, AKR1C2 |
| 37 | Li W,  PMID: 29228771 | In Vitro | Nrf2 |
| 38 | Chen S,  PMID: 29207190 | In Vitro | NFκB, 4EBP1, p4EBP1, mTOR, pmTOR, AKT, pATK |
| 39 | Ramya PVS,  PMID: 29174816 | In Vitro | Tubulin |
| 40 | Zhang H,  PMID: 29172709 | In Vitro | miR7705p, miR1247 |
| 41 | Liu T,  PMID: 28843521 | In Vitro,  In Vivo (mouse) | miR145, lncRNAROR, Oct4 |
| 42 | Rivera M,  PMID: 28628644 | In Vitro | ER stress-mediated apoptosis |
| 43 | Wang R,  PMID: 28528814 | In Vivo (mouse) | Malat1-short interfering RNA, AR-v7 (androgen receptor) degradation enhancer |
| 44 | Liu J,  PMID: 28391715 | In Vitro | miR143, miR145, ATG2B |
| 45 | Cao H,  PMID: 28391351 | In Vitro | FOXD3, miR143, PGK1 |
| 46 | Rodriguez-Garcia A,  PMID: 28391184 | In Vitro | Thioredoxin 1 |
| 47 | Yang C,  PMID: 28243065 | In Vitro | TfR1, IRP1 |
| 48 | Banerjee S,  PMID: 28199187 | In Vitro | RTKs, PI3K, pAKT, NFκB, p53, COX2 |
| 49 | Yang J,  PMID: 28120490 | In Vitro | Notch1 |
| 50 | Sri Ramya PV,  PMID: 28038323 | In Vitro | Tubulin, mitochondrial membrane potential |
| 51 | Chen M,  PMID: 27990666 | In Vitro,  In Vivo (mouse) | ROS |
| 52 | Fehl DJ,  PMID: 27865863 | In Vitro,  In Vivo (mouse) | Bclxl, NFκB, STAT1 |
| 53 | Sha J,  PMID: 27657825 | In Vitro | Notch1, CyclinD1, CDK2, p21, p27, p53, Bcl2, Caspase3, Caspase9 |
| 54 | Zhang X,  PMID: 27543391 | In Vitro | Apoptosis, G0/G1 phase |
| 55 | Mandalapu D,  PMID: 27496212 | In Vitro | PCNA, pAkt, Bax, Bcl2 |
| 56 | Nagahama K,  PMID: 27476814 | In Vitro | pH-responsive endosomal disrupting activity |
| 57 | Jayaprakasha GK,  PMID: 27404761 | In Vitro | p53, bax, Bcl2, cytochrome-c |
| 58 | Zhou DY,  PMID: 27313760 | In Vitro | Androgen receptor |
| 59 | Wang R,  PMID: 27233475 | In Vitro | Androgen receptor F876L |
| 60 | Sharma V,  PMID: 27132804 | In Vitro | DNA methyltransferase, androgen receptor |
| 61 | Wen S,  PMID: 27045473 | In Vitro | EZH2, STAT3, Akt |
| 62 | Li W,  PMID: 26991801 | In Vitro | Nrf2, keap1, CpG demethylation |
| 63 | Wen S,  PMID: 26894509 | In Vitro | Fatty acid synthase |
| 64 | Hu HJ,  PMID: 26893768 | In Vitro | cMet |
| 65 | Wang R,  PMID: 26827161 | In Vitro | Apoptosis, G0/G1 phase |
| 66 | Kang M,  PMID: 26718024 | In Vitro | Apoptosis |
| 67 | Huang H,  PMID: 26630272 | In Vitro,  In Vivo (mouse) | NFκB, Bcl2, pAkt, pERK1/2 |
| 68 | Zhang X,  PMID: 26546056 | In Vitro | ROS |
| 69 | Du Y,  PMID: 26499200 | In Vitro | MAOA, mTOR, HIF1α, CXCR4, IL6, ROS |
| 70 | Yang J,  PMID: 26464676 | In Vivo (mouse) | Bcl2, Bax |
| 71 | Hong JH,  PMID: 26366279 | In Vivo (mouse) | PSA, androgen receptor |
| 72 | Chen QH,  PMID: 26341135 | In Vitro | Anti-proliferation |
| 73 | Yan J,  PMID: 26203689 | In Vitro,  In Vivo (mouse) | Growth inhibition |
| 74 | Lee WJ,  PMID: 26013662 | In Vitro,  In Vivo (mouse) | CHOP, Bip/GRP78, LC3II, ROS |
| 75 | Li J,  PMID: 25971429 | In Vitro | ERK1/2, SAPK, JNK, p65, MUC1C |
| 76 | Li Q,  PMID: 25728027 | In Vitro | Free radical |
| 77 | Linnewiel-Hermoni K,  PMID: 25711533 | In Vitro | EpRE/ARE, androgen receptor |
| 78 | Wu M,  PMID: 25704883 | In Vitro,  In Vivo (mouse) | Calmodulin, androgen receptor |
| 79 | Fernandez-Martinez AB,  PMID: 25446255 | In Vitro | VIP, COX2, PGE2, VEGF |
| 80 | Eom DW,  PMID: 25441423 | In Vitro | p21 |
| 81 | Wang P,  PMID: 25243063 | In Vitro | Bax, Bcl2, NFκB, PI3K, Akt, Stat3 |
| 82 | Mathur A,  PMID: 25121735 | In Vitro,  In Vivo (mouse) | Akt, eIF2α, ATF4, CHOP, TRIB3 |
| 83 | Zhou DY,  PMID: 25060817 | In Vitro | Androgen receptor |
| 84 | Yallapu MM,  PMID: 25028336 | In Vitro,  In Vivo (mouse) | Androgen receptor, βcatenin, STAT3, Akt, Mcl1, BclxL, PARP, miR21, miR205 |
| 85 | Dorai T,  PMID: 24949215 | In Vitro,  In Vivo (mouse) | BMP7, TGFβ |
| 86 | Yu XL,  PMID: 24606484 | In Vitro,  In Vivo (mouse) | Id1 |
| 87 | Luo C,  PMID: 24297639 | In Vitro,  In Vivo (mouse) | ROS |
| 88 | Gupta A,  PMID: 24216994 | In Vitro | MMP9, VEGF, angiostatin |
| 89 | Piccolella M,  PMID: 24184124 | In Vitro | ERβ |
| 90 | Harada T,  PMID: 24160991 | In Vitro | Growth inhibition |
| 91 | Botchkina GI,  PMID: 24086245 | In Vivo (mouse) | p21, p53 |
| 92 | Zhou DY,  PMID: 23985704 | In Vitro | Akt, Erk1/2 |
| 93 | Yang CH,  PMID: 23940701 | In Vitro,  In Vivo (mouse) | NFκB, miR21 |
| 94 | Lin TH,  PMID: 23928703 | In Vitro,  In Vivo (mouse) | pSTAT3, CCL2, PIAS3, STAT3 |
| 95 | Guo H,  PMID: 23875250 | In Vitro | NFκB, IFκB, cJun, androgen receptor |
| 96 | Lin TH,  PMID: 23687298 | In Vitro,  In Vivo (mouse) | MMP9 |
| 97 | Wei X,  PMID: 23564771 | In Vitro | NFκB, ERK1/2 |
| 98 | Cheng TS,  PMID: 23466486 | In Vitro,  In Vivo (mouse) | EGF, heregulin, matrix metalloproteinase 9, matripase |
| 99 | Kang D,  PMID: 23353183 | In Vitro | ROS, JNK, p38, ERK, Akt |
| 100 | Lai KP,  PMID: 23219429 | In Vitro,  In Vivo (mouse) | AR-Mdm2 (androgen receptor) |
| 101 | Chen SS,  PMID: 23200064 | In Vitro | IDO1 |
| 102 | Killian PH,  PMID: 23042094 | In Vitro,  In Vivo (mouse) | NFκB, CXCL1, CXCL2, COX2, SPARC, EFEMP |
| 103 | Bao B,  PMID: 22952749 | In Vitro | VEGF, IL6, Nanog, Oct4, EZH2, miR21 |
| 104 | Hung CM,  PMID: 22849866 | In Vitro | AMPK, FASN, ACC, caspase3, EGFR, PP2a, SHP2 |
| 105 | Wei X,  PMID: 22844370 | In Vitro | NFκB |
| 106 | Lee SO,  PMID: 22831834 | In Vitro,  In Vivo (mouse) | Stem/progenitor cells |
| 107 | Pramanik D,  PMID: 22791660 | In Vitro,  In Vivo (mouse) | MDR phenotype |
| 108 | Guo X,  PMID: 22711297 | In Vitro | Mcl1, AIF, focal adhesion kinase |
| 109 | Shi Q,  PMID: 22672984 | In Vitro | Pseudopodia |
| 110 | Ni X,  PMID: 22552297 | In Vitro | Caspase3, MMP2 |
| 111 | Wei X,  PMID: 22551677 | In Vitro | Apoptosis |
| 112 | Sundram V, PMID: 22523587 | In Vitro,  In Vivo (mouse) | PKD1, βcatenin |
| 113 | Teiten MH,  PMID: 22475723 | In Vitro | PPP2R1A, RBM17, DDX39, HMGB1, NPM1, NPM1, FKBP4/FKBP52, miR141, miR152, miR183 |
| 114 | Yamashita S,  PMID: 22355276 | In Vitro,  In Vivo (mouse) | AR3, fAR (androgen receptor) |
| 115 | Shah S,  PMID: 22258452 | In Vitro,  In Vivo (mouse) | p300, GATA2, FOXA1, AR histone modification (androgen receptor) |
| 116 | Farooqi AA,  PMID: 22070051 | In Vitro | PDGFR, EGFR |
| 117 | Shu L,  PMID: 21938566 | In Vitro | HDAC, Neurog1, CpG demethylation |
| 118 | Chung LC,  PMID: 21936051 | In Vitro | HIF1α, PSA |
| 119 | Liu S,  PMID: 21823017 | In Vitro | NFκB, AP1 |
| 120 | Fajardo AM,  PMID: 21796654 | In Vitro | ROS, Nrf2, NAD(P)H quinone-oxidoreductase-1, aldoketoreductase1C1, androgen receptor |
| 121 | Khor TO,  PMID: 21787756 | In Vitro | Nrf2 |
| 122 | Li Y,  PMID: 21680704 | In Vitro | TMPRSS2, ERG, Wnt |
| 123 | Teiten MH,  PMID: 21240460 | In Vitro | Tcf4, CBP, p300, Wnt, βcatenin, Tcf4, cyclinD1, cmyc |
| 124 | Lamson DW,  PMID: 21194250 | In Vitro | VC:VK₃ |
| 125 | Ide H,  PMID: 21134073 | In Vitro | ATM, histone H2AX, Chk2, p53, PARP |
| 126 | Wan SB,  PMID: 20818481 | In Vitro | Proteasome inhibitors |
| 127 | Choi HY,  PMID: 20680030 | In Vitro | CyclinD1, cmyc, βcatenin, T-cell factor, akt, glycogen synthase kinase-3β |
| 128 | Slusarz A,  PMID: 20395211 | In Vitro,  In Vivo (mouse) | Hedgehog |
| 129 | Hilchie AL,  PMID: 20358476 | In Vitro | AIF, p38, MAPK, JNK, caspase3, caspase8, caspase9 |
| 130 | Fernandez-Martinez AB,  PMID: 19772879 | In Vitro,  In Vivo (mouse) | VIP, VPAC(1), VEGF, COX2, MMP2, MMP9 |
| 131 | Zhou J,  PMID: 19725582 | In Vitro | Androgen receptor |
| 132 | Lin L,  PMID: 19558577 | In Vitro | Akt, HER2/neu, STAT3 |
| 133 | Piantino CB,  PMID: 19538771 | In Vitro | Apoptosis |
| 134 | Herman JG,  PMID: 19360344 | In Vitro | CCL2, PKC, MMP9 |
| 135 | Narayanan NK,  PMID: 19326431 | In Vitro,  In Vivo (mouse) | PTEN, pAkt, cyclin D1 |
| 136 | Andrzejewski T,  PMID: 19192720 | In Vivo (mouse) | pAkt, NFκB |
| 137 | Fernandez-Martinez AB,  PMID: 19189304 | In Vitro | VIP, NFκB, MMP2, MMP9 |
| 138 | Valentini A,  PMID: 19113979 | In Vitro | ROS, JNK, GSTp1, Bax, Bcl2 |
| 139 | Santel T,  PMID: 18946510 | In Vitro | Glo1 |
| 140 | Yu S,  PMID: 18790744 | In Vitro | Akt, mTOR |
| 141 | Thangapazham RL,  PMID: 18719366 | In Vitro | EGFR, ERBB2 |
| 142 | Thomas SL,  PMID: 18682687 | In Vitro | HIF |
| 143 | Tsui KH,  PMID: 18676361 | In Vitro | R1881, IL6 |
| 144 | Barve A,  PMID: 18437538 | In Vivo (mouse) | Akt |
| 145 | Deng G,  PMID: 18390174 | In Vitro | VEGF |
| 146 | Shankar S,  PMID: 18226269 | In Vivo (mouse) | TRAILR1/DR4, TRAILR2/DR5, Bax, Bak, p21, WAF1, p27, KIP1, NFκB, cyclinD1, VEGF, uPA, MMP2, MMP9, Bcl2, BclXL, EGFR2 |
| 147 | Srivastava RK,  PMID: 18156803 | In Vitro | CDK, p16(/INK4a), p21(/WAF1/CIP1), p27(/KIP1), cyclinE, cyclinD1, Rb |
| 148 | Shankar S,  PMID: 17916240 | In Vitro | Bcl2, BclXL, survivin, XIAP, Bax, Bak, PUMA, Bim, Noxa, TRAILR1/DR4, TRAILR2/DR5, caspase3, caspase9, capillary tube formation |
| 149 | Shankar S,  PMID: 17332930 | In Vitro | Akt, Smac, p53 |
| 150 | Li M,  PMID: 17332326 | In Vitro,  In Vivo (mouse) | MDM2, p21, Waf1/CIP1 |
| 151 | Zhang HN,  PMID: 17303007 | In Vitro | NKX3.1 |
| 152 | Shi P,  PMID: 17290611 | In Vitro | Maspin |
| 153 | Deeb D,  PMID: 17289836 | In Vitro | Akt, NFκB, Bcl2, BclxL, XIAP |
| 154 | Nonn L,  PMID: 17151092 | In Vitro | MKP5 |
| 155 | Guo H,  PMID: 17147887 | In Vitro | IκBα |
| 156 | Marcu MG,  PMID: 16787365 | In Vitro | p300, CBP, HAT inhibitors |
| 157 | Guo H,  PMID: 16519151 | In Vitro | G2/M phase |
| 158 | Deeb DD,  PMID: 16471035 | In Vitro | Annexin V, DNA fragmentation, procaspase3, procaspase8, procaspase9, Bid, cytochrome-c, NFκB |
| 159 | Lin L,  PMID: 16427289 | In Vitro | Androgen receptor |
| 160 | Khor TO,  PMID: 16423986 | In Vivo (mouse) | Akt, NFκB |
| 161 | Hong JH,  PMID: 16389264 | In Vitro,  In Vivo (mouse) | MMP2, MMP9 |
| 162 | Yang L,  PMID: 16342680 | In Vitro | PSA, luciferase, androgen receptor |
| 163 | Kim JH,  PMID: 16299382 | In Vitro | EGFR, Akt, NFκB |
| 164 | Polytarchou C,  PMID: 16199533 | In Vitro | HARP, AP1 |
| 165 | Collado B,  PMID: 15921770 | In Vitro | VEGF |
| 166 | Adams BK,  PMID: 15711178 | In Vitro | Caspase3, phosphatidylserine, DNA fraction, redox |
| 167 | Shenouda NS,  PMID: 15489213 | In Vitro | G2/M phase |
| 168 | Deeb D,  PMID: 15252141 | In Vitro | NFκB, IκBα |
| 169 | Holy J,  PMID: 15236356 | In Vitro | CB, PKC, microfilaments |
| 170 | Dorai T,  PMID: 15129424 | In Vitro | EGFR, CSF1R, a1 |
| 171 | Chendil D,  PMID: 14985701 | In Vitro | Cytochrome-c, caspase9, caspase3, TNFα, NFκB |
| 172 | Kumar AP,  PMID: 12869308 | In Vitro | Akt, NFκB, p65 |
| 173 | Ghosh J,  PMID: 12859962 | In Vitro | Jnk, 5-lipoxygenase |
| 174 | Park JI,  PMID: 12853969 | In Vitro | TGFβ1, cJun phosphorylation, IL6 |
| 175 | Chaudhary LR,  PMID: 12682902 | In Vitro | Akt |
| 176 | Deeb D,  PMID: 12533677 | In Vitro | Procaspase3, procaspase8, procaspase9, Bid, cytochrome-c |
| 177 | Dorai T,  PMID: 12497104 | In Vitro | Bcl2, bclxL, androgen receptor |
| 178 | Mukhopadhyay A, PMID: 12483537 | In Vitro | CyclinD1, lactacystin |
| 179 | Nakamura K,  PMID: 12239622 | In Vitro | Androgen receptor, AP1, CREB, CPBP, NFκB |
| 180 | Hour TC,  PMID: 11967955 | In Vitro | p21, WAF1/CIP1, C/EBPβ, NFκB, TNFα |
| 181 | Mukhopadhyay A,  PMID: 11753638 | In Vitro | NFκB, TNF, Bcl2, BclxL, procaspase3, procaspase8, AP1 |
| 182 | Dorai T,  PMID: 11398177 | In Vivo (mouse) | Apoptosis, microvessel density |
| 183 | Imaida K,  PMID: 11238188 | In Vivo (rat) | None reported |
| 184 | Dorai T,  PMID: 10851300 | In Vitro | EGFR |
