# Supplementary material for: Effects of curcumin and ursolic acid in prostate cancer: A systematic review
Source: Urologia. 2023 Sep 30;91(1):90–106. doi: 10.1177/03915603231202304 (PMC10976464; doi:10.1177/03915603231202304)
Supplement: sj-docx-4-urj-10.1177_03915603231202304 – Supplemental material for Effects of curcumin and ursolic acid in prostate cancer: A systematic review [file sj-docx-4-urj-10.1177_03915603231202304.docx]

**Supplementary Table 4.** Word cloud summary table (corresponding to Figure 5a).

| Mechanistic Pathway/Effect | Count | Weighted Percent | Percent of Pathway/Effect Outcome Studies |
| --- | --- | --- | --- |
| nfκb | 25 | 4.42% | 14.5% |
| androgen | 19 | 3.36% | 11.0% |
| akt | 18 | 3.18% | 10.4% |
| bcl2 | 12 | 2.12% | 6.9% |
| mmp9 | 8 | 1.41% | 4.6% |
| p21 | 8 | 1.41% | 4.6% |
| ros | 8 | 1.41% | 4.6% |
| apoptosis | 7 | 1.24% | 4.0% |
| bax | 7 | 1.24% | 4.0% |
| bclxl | 7 | 1.24% | 4.0% |
| caspase3 | 7 | 1.24% | 4.0% |
| p53 | 7 | 1.24% | 4.0% |
| psa | 7 | 1.24% | 4.0% |
| stat3 | 7 | 1.24% | 4.0% |
| vegf | 7 | 1.24% | 4.0% |
| cyclind1 | 6 | 1.06% | 3.5% |
| egfr | 6 | 1.06% | 3.5% |
| jnk | 6 | 1.06% | 3.5% |
| mmp2 | 6 | 1.06% | 3.5% |
| pakt | 5 | 0.88% | 2.9% |
| phase | 5 | 0.88% | 2.9% |
| βcatenin | 5 | 0.88% | 2.9% |
| ap1 | 4 | 0.71% | 2.3% |
| caspase9 | 4 | 0.71% | 2.3% |
| cip1 | 4 | 0.71% | 2.3% |
| cox2 | 4 | 0.71% | 2.3% |
| cytochrome | 4 | 0.71% | 2.3% |
| il6 | 4 | 0.71% | 2.3% |
| nrf2 | 4 | 0.71% | 2.3% |
| waf1 | 4 | 0.71% | 2.3% |

Top 30 most common (count ≥4) molecular and cellular pathways from articles reporting on the pathways and effects of **curcumin** (n=173) in prostate cancer. Weighted Percentage is the frequency of the word relative to the total words counted.
