# Supplementary material for: Effects of curcumin and ursolic acid in prostate cancer: A systematic review
Source: Urologia. 2023 Sep 30;91(1):90–106. doi: 10.1177/03915603231202304 (PMC10976464; doi:10.1177/03915603231202304)
Supplement: sj-docx-5-urj-10.1177_03915603231202304 – Supplemental material for Effects of curcumin and ursolic acid in prostate cancer: A systematic review [file sj-docx-5-urj-10.1177_03915603231202304.docx]

**Supplementary Table 5.** Reviewed articles reporting on the pathways and effects of **ursolic acid** (n=24) in prostate cancer**.**

|  | Study ID | Participants | Mechanistic Pathway |
| --- | --- | --- | --- |
| 7 | Lodi A,  PMID: 29202102 | In Vitro,  In Vivo (mouse) | STAT3, Src, mTORC1, AMPK |
| 185 | Song Y,  PMID: 31600936 | Unknown | MAPK8, IL6, VEGFA, STAT3, JUN, CXCL8, IL1B, MMP9, CCL2, RELA, CREB1 |
| 186 | Mu D,  PMID: 29435058 | In Vitro | ROCK, PTEN, cofilin1 |
| 187 | Wang C,  PMID: 29383876 | In Vitro | Setd7, Nrf2, ARE |
| 188 | Gai WT,  PMID: 27698874 | In Vitro | ROCK, PTEN, coflin1, caspase3, caspase9 |
| 189 | Pedada SR,  PMID: 26907155 | In Vitro,  In Vivo (rat) | sPLA2 |
| 190 | Meng Y,  PMID: 26503559 | In Vitro,  In Vivo (mouse) | PI3K, Akt, mTOR |
| 191 | Mallavadhania UV,  PMID: 25632459 | In Vitro | Cytotoxic |
| 192 | Kim HI,  PMID: 25088993 | In Vitro | Prostate-specific antigen promoter activity |
| 193 | Park JH,  PMID: 24399733 | In Vitro | Wnt5, βcatenin, caspase9 |
| 194 | Shin SW,  PMID: 23247106 | In Vitro | TRAIL, CHOP, DR5, PTEN |
| 195 | Limami Y,  PMID: 22521508 | In Vitro | P2Y(2) Receptor, COX2 |
| 196 | Liu YW,  PMID: 22429052 | In Vitro | Anti-inflammatory |
| 197 | Shanmugam MK,  PMID: 22427843 | In Vivo (mouse) | NFκB, STAT3, AKT, IKKα/β, TNFα, IL6, cyclin D1, COX2, caspase3 |
| 198 | Koh SJ,  PMID: 22239065 | In Vitro | Caspase3 |
| 199 | Shin SW,  PMID: 22178132 | In Vitro | Akt, mTOR, Beclin1 |
| 200 | Shanmugam MK,  PMID: 21480220 | In Vitro,  In Vivo (mouse) | CXCR4, NFκB |
| 201 | Shanmugam MK,  PMID: 21465181 | In Vitro,  In Vivo (mouse) | TNFα, NFκB, STAT3 |
| 202 | Kondo M,  PMID: 21351105 | In Vitro | MMP2, MMP9 |
| 203 | Kwon SH,  PMID: 20943386 | In Vitro | Caspase3, Caspase8, Caspase9, Bax, Bcl2 |
| 204 | Zhang Y,  PMID: 20146252 | In Vitro | Caspase8, Caspase9, Akt, Fas |
| 205 | Zhang YX,  PMID: 20052671 | In Vitro | Caspase9, Bcl2 |
| 206 | Zhang YX,  PMID: 19545597 | In Vitro | caspase3, JNK, Bcl2 |
| 207 | Kassi E,  PMID: 17516089 | In Vitro | Bcl2 |
