# Supplementary material for: Effects of curcumin and ursolic acid in prostate cancer: A systematic review
Source: Urologia. 2023 Sep 30;91(1):90–106. doi: 10.1177/03915603231202304 (PMC10976464; doi:10.1177/03915603231202304)
Supplement: sj-docx-6-urj-10.1177_03915603231202304 – Supplemental material for Effects of curcumin and ursolic acid in prostate cancer: A systematic review [file sj-docx-6-urj-10.1177_03915603231202304.docx]

**Supplementary Table 6.** Word cloud summary table (corresponding to Figure 5b).

| Mechanistic Pathway/Effect | Count | Weighted Percentage | Percent of Pathway/Effect Outcome Studies |  |
| --- | --- | --- | --- | --- |
| caspase3 | 5 | 6.41% | 20.8% | |
| caspase9 | 5 | 6.41% | 20.8% | |
| akt | 4 | 5.13% | 16.7% | |
| bcl2 | 4 | 5.13% | 16.7% | |
| stat3 | 4 | 5.13% | 16.7% | |
| nfκb | 3 | 3.85% | 12.5% | |
| pten | 3 | 3.85% | 12.5% | |
| caspase8 | 2 | 2.56% | 8.3% | |
| cox2 | 2 | 2.56% | 8.3% | |
| il6 | 2 | 2.56% | 8.3% | |
| mmp9 | 2 | 2.56% | 8.3% | |
| mtor | 2 | 2.56% | 8.3% | |
| rock | 2 | 2.56% | 8.3% | |
| tnfα | 2 | 2.56% | 8.3% | |

Top 14 most common (count ≥2) molecular and cellular pathways from articles reporting on the pathways and effects of **ursolic acid** (n=24) in prostate cancer. Weighted Percentage is the frequency of the word relative to the total words counted.
