# Supplementary material for: Effects of curcumin and ursolic acid in prostate cancer: A systematic review
Source: Urologia. 2023 Sep 30;91(1):90–106. doi: 10.1177/03915603231202304 (PMC10976464; doi:10.1177/03915603231202304)
Supplement: sj-docx-7-urj-10.1177_03915603231202304 – Supplemental material for Effects of curcumin and ursolic acid in prostate cancer: A systematic review [file sj-docx-7-urj-10.1177_03915603231202304.docx]

**Supplementary Table 7.** Word cloud summary table (corresponding to Figure 5c).

| Mechanistic Pathway/Effect | Count | Weighted Percentage | Percent of Pathway/Effect Outcome Studies |
| --- | --- | --- | --- |
| nfκb | 28 | 4.35% | 14.2% |
| akt | 22 | 3.42% | 11.2% |
| androgen | 19 | 2.95% | 9.6% |
| bcl2 | 16 | 2.48% | 8.1% |
| caspase3 | 12 | 1.86% | 6.1% |
| stat3 | 11 | 1.71% | 5.6% |
| mmp9 | 10 | 1.55% | 5.1% |
| caspase9 | 9 | 1.40% | 4.6% |
| bax | 8 | 1.24% | 4.1% |
| p21 | 8 | 1.24% | 4.1% |
| ros | 8 | 1.24% | 4.1% |
| apoptosis | 7 | 1.09% | 3.6% |
| bclxl | 7 | 1.09% | 3.6% |
| jnk | 7 | 1.09% | 3.6% |
| mmp2 | 7 | 1.09% | 3.6% |
| p53 | 7 | 1.09% | 3.6% |
| psa | 7 | 1.09% | 3.6% |
| vegf | 7 | 1.09% | 3.6% |
| cox2 | 6 | 0.93% | 3.0% |
| cyclind1 | 6 | 0.93% | 3.0% |
| egfr | 6 | 0.93% | 3.0% |
| il6 | 6 | 0.93% | 3.0% |
| βcatenin | 6 | 0.93% | 3.0% |
| mtor | 5 | 0.78% | 2.5% |
| nrf2 | 5 | 0.78% | 2.5% |
| pakt | 5 | 0.78% | 2.5% |
| phase | 5 | 0.78% | 2.5% |
| ap1 | 4 | 0.62% | 2.0% |
| cip1 | 4 | 0.62% | 2.0% |
| cyclin | 4 | 0.62% | 2.0% |
| cytochrome | 4 | 0.62% | 2.0% |
| pi3k | 4 | 0.62% | 2.0% |
| pten | 4 | 0.62% | 2.0% |
| tnfα | 4 | 0.62% | 2.0% |
| waf1 | 4 | 0.62% | 2.0% |

Top 35 most common (count ≥4) molecular and cellular pathways from articles reporting on the pathways and effects (n=197) of both **curcumin** (n=173) and **ursolic acid** (n=24) in prostate cancer. Weighted Percentage is the frequency of the word relative to the total words counted.
