# Supplementary material for: Effects of curcumin and ursolic acid in prostate cancer: A systematic review
Source: Urologia. 2023 Sep 30;91(1):90–106. doi: 10.1177/03915603231202304 (PMC10976464; doi:10.1177/03915603231202304)
Supplement: sj-docx-8-urj-10.1177_03915603231202304 – Supplemental material for Effects of curcumin and ursolic acid in prostate cancer: A systematic review [file sj-docx-8-urj-10.1177_03915603231202304.docx]

**Supplementary Table 8.** Reviewed articles reporting on the absorption and bioavailability of **curcumin** (n=58) in prostate cancer**.**

|  | Study ID | Participants | Delivery Method |
| --- | --- | --- | --- |
| 14 | Greil R,  PMID: 30074076 | In Vivo (human) | Liposomal curcumin |
| 27 | Belluti S,  PMID: 30577600 | In Vitro | Curcumin analog |
| 29 | Vellampatti S,  PMID: 30297802 | In Vitro | Cu2+/Ni2+-curcumin |
| 32 | Plyduang T,  PMID: 30062740 | In Vitro | Polyacetal-curcumin-diethylstilbestrol |
| 38 | Chen S,  PMID: 29207190 | In Vitro | Curcumin analog |
| 57 | Jayaprakasha GK,  PMID: 27404761 | In Vitro | Nanoencapsulated curcumin (NEC) |
| 73 | Yan J,  PMID: 26203689 | In Vitro,  In Vivo (mouse) | Curcumin co-encapsulated lipid-polymer hybrid nanoparticles D(TX-CUR-LPNs) |
| 76 | Li Q,  PMID: 25728027 | In Vitro | Curcumin analog |
| 81 | Wang P,  PMID: 25243063 | In Vitro | Curcumin mixture |
| 84 | Yallapu MM,  PMID: 25028336 | In Vitro,  In Vivo (mouse) | Novel poly(lactic-co-glycolic acid)-curcumin nanoparticles (PLGA-CUR NPs) |
| 87 | Luo C,  PMID: 24297639 | In Vitro,  In Vivo (mouse) | Curcumin analog |
| 90 | Harada T,  PMID: 24160991 | In Vitro | Delivery of curcumin by 66γCD2ur |
| 135 | Narayanan NK,  PMID: 19326431 | In Vitro,  In Vivo (mouse) | Liposome encapsulated curcumin |
| 208 | Ma Q,  PMID: 31819373 | In Vitro | Curcumin nanoliposomes |
| 209 | Jahanshahi M,  PMID: 31678390 | In Vitro | Curcumin charged nanoparticles |
| 210 | Li H,  PMID: 31578087 | In Vitro,  In Vivo (mouse) | Single-walled carbon nanotubes |
| 211 | Gracia E,  PMID: 31569529 | In Vivo (mouse) | Curcumin-impregnated via biodegradable poly(lactic-co-glycolic) acid (PLGA) nanosphere |
| 212 | Puiggali­-Jou A,  PMID: 31430890 | In Vitro | Curcumin-loaded nanoparticles or fibers |
| 213 | Chu PY,  PMID: 31192580 | In Vitro,  In Vivo (mouse) | Curcumin nanoparticle system |
| 214 | Ke X,  PMID: 31009696 | In Vitro | Flash nanocomplexation (FNC) |
| 215 | Bessone F,  PMID: 30654342 | In Vitro | Curcuminpolymeric nanobubbles |
| 216 | O'Connor NA,  PMID: 30555179 | In Vitro | Curcumin with a self-degrading polymer |
| 217 | Meng X,  PMID: 30370853 | In Vitro | Curcumin analog |
| 218 | Patanapongpibul M,  PMID: 30121214 | In Vitro,  In Vivo (rat) | Curcumin analog |
| 219 | Gdowski A,  PMID: 29433518 | In Vitro | Nanolipomer formulation |
| 220 | Cheng MA,  PMID: 29203251 | In Vitro,  In Vivo (mouse) | Dimethylcurcumin dissolved in FDA-approved solvents, including DMSO, PEG-400 |
| 221 | Azandeh SS,  PMID: 29201078 | In Vitro | Encapsulation of Cur in PLGA (poly lactic-coglycolic acid) nanospheres |
| 222 | Zhang X,  PMID: 28601720 | In Vitro,  In Vivo (rat) | Curcumin analog |
| 223 | Guan YB,  PMID: 28585133 | In Vitro | Curcumin nanoemulsions |
| 224 | Saralkar P,  PMID: 28397161 | In Vitro | Drug-loaded nanoparticles |
| 225 | Yan J,  PMID: 28122302 | In Vitro | Curcumin nanoparticle delivery |
| 226 | Klippstein R,  PMID: 27863661 | In Vitro | Curcumin nanocapsules |
| 226 | Aldahoun MA, PMID: 27137748 | In Vitro | Curcumin nanocapsules |
| 228 | de Oliveira LF,  PMID: 26930039 | In Vitro | Amino-functionalized silica nanoparticles loaded with curcumin |
| 229 | Adahoun MA,  PMID: 26747522 | In Vitro | Toxicity for nanocurcumin |
| 230 | Shukla P,  PMID: 26000396 | In Vitro | Curcumin encapsulation in nanoemulsion |
| 231 | Wang R,  PMID: 25961334 | In Vitro | Curcumin analog |
| 232 | Thangavel S,  PMID: 25912409 | In Vitro,  In Vivo (mouse) | Entrapment of both curcumin in hydrophobic core liposome of RNP(N) |
| 233 | Citalingam K,  PMID: 25690296 | In Vitro | Curcumin analog |
| 234 | Zhou D,  PMID: 25451846 | In Vitro | Curcumin analog |
| 235 | Samaan N,  PMID: 24531225 | In Vitro | Curcumin analog |
| 236 | Rao W,  PMID: 24516867 | In Vitro | Polymeric nanoparticle-encapsulated curcumin (nCCM) |
| 237 | Soh SF,  PMID: 24042123 | In Vivo (mouse) | Curcumin analog |
| 238 | Boztas AO,  PMID: 23730903 | In Vitro | Encapsulating curcumin in poly(β-cyclodextrin triazine) (PCDT) |
| 239 | Salehi P,  PMID: 23565869 | In Vitro | N-isopropylacrylamide (NIPAAM)/N-vinyl-2-pyrrolidone (VP)/Polyethylene glycol monoacrylate (PEG-A) polymeric nanoparticles encapsulating curcumin |
| 240 | Aditya NP,  PMID: 23362941 | In Vitro | Curcumin loaded nanostructured lipid carriers (NLCs) |
| 241 | Yallapu MM,  PMID: 21892919 | In Vitro | Curcumin loaded cellulose nanoparticles (cellulose-CUR) |
| 242 | Rejinold NS,  PMID: 21870456 | In Vitro | Curcumin loaded fibrinogen nanoparticles (CRC-FNPs) |
| 243 | Agashe H,  PMID: 21779150 | In Vitro,  In Vivo (rat) | Curcumin liposome |
| 244 | Yallapu MM,  PMID: 20572274 | In Vitro | Cyclodextrin polymer and curcumin (PCD/CUR) formulation |
| 245 | Yallapu MM,  PMID: 20456930 | In Vitro | Beta-cyclodextrin-curcumin self-assembly |
| 246 | Mukerjee A,  PMID: 19846921 | In Vitro | Curcumin loaded PLGA nanospheres |
| 247 | Fuchs JR,  PMID: 19249204 | In Vitro | Curcumin analog |
| 248 | Thangapazham RL,  PMID: 18425340 | In Vitro | Curcumin liposomes from dimyristoyl phosphatidyl choline (DMPC) and cholesterol |
| 249 | Abas F,  PMID: 17137104 | In Vitro | Curcumin analog |
| 250 | Lin L,  PMID: 16789753 | In Vitro | Curcumin analog |
| 251 | Kachadourian R,  PMID: 16781454 | In Vitro | Curcumin potentiation |
| 252 | Ohtsu H,  PMID: 12408714 | In Vitro | Curcumin analog |
