# Supplementary material for: Effects of curcumin and ursolic acid in prostate cancer: A systematic review
Source: Urologia. 2023 Sep 30;91(1):90–106. doi: 10.1177/03915603231202304 (PMC10976464; doi:10.1177/03915603231202304)
Supplement: sj-docx-9-urj-10.1177_03915603231202304 – Supplemental material for Effects of curcumin and ursolic acid in prostate cancer: A systematic review [file sj-docx-9-urj-10.1177_03915603231202304.docx]

**Supplementary Table 9.** Reviewed articles reporting on the absorption and bioavailability of **ursolic acid** (n=4) in prostate cancer**.**

|  | Study ID | Participants | Delivery Method |
| --- | --- | --- | --- |
| 197 | Shanmugam MK,  PMID: 22427843 | In Vivo (mouse) | Ursolic acid serum levels |
| 201 | Shanmugam MK,  PMID: 21465181 | In Vitro,  In Vivo (mouse) | Ursolic acid uptake |
| 253 | Caldeira de Araujo Lopes S,  PMID: 23984367 | In Vitro | Ursolic acid liposomes |
| 254 | Murphy BT,  PMID: 12769521 | In Vitro | Ursolic acid derivative |
